# Innovative Technologies in the Neurorehabilitation of Traumatic Brain Injury: A Systematic Review

**DOI:** 10.3390/brainsci12121678

**Published:** 2022-12-07

**Authors:** Mirjam Bonanno, Rosaria De Luca, Alessandro Marco De Nunzio, Angelo Quartarone, Rocco Salvatore Calabrò

**Affiliations:** 1 IRCCS Centro Neurolesi “Bonino-Pulejo”, Via Palermo, SS 113, C. da Casazza, 98124 Messina, Italy; 2 Department of Research and Development, LUNEX International University of Health, Exercise and Sports, Avenue du Parc des Sports, 50, 4671 Differdange, Luxembourg

**Keywords:** robotic device, virtual reality, innovations in neurorehabilitation, traumatic brain injury

## Abstract

Motor and cognitive rehabilitation in individuals with traumatic brain injury (TBI) is a growing field of clinical and research interest. In fact, novel rehabilitative approaches allow a very early verticalization and gait training through robotic devices and other innovative tools boosting neuroplasticity, thanks to the high-intensity, repetitive and task-oriented training. In the same way, cognitive rehabilitation is also evolving towards advanced interventions using virtual reality (VR), computer-based approaches, telerehabilitation and neuromodulation devices. This review aimed to systematically investigate the existing evidence concerning the role of innovative technologies in the motor and cognitive neurorehabilitation of TBI patients. We searched and reviewed the studies published in the Cochrane Library, PEDro, PubMed and Scopus between January 2012 and September 2022. After an accurate screening, only 29 papers were included in this review. This systematic review has demonstrated the beneficial role of innovative technologies when applied to cognitive rehabilitation in patients with TBI, while evidence of their effect on motor rehabilitation in this patient population is poor and still controversial.

## 1. Introduction

Traumatic Brain Injury (TBI) is non-progressive damage to the brain followed by a violent and rapid external force applied to the skull. TBI affects around 64–74 million persons each year worldwide and causes a variety of physical, motor, speech, and cognitive deficits that can have a long-term detrimental impact [1,2]. In fact, beyond motor impairment, attention, memory, affectivity, behaviour and executive dysfunctions may occur after the brain damage involving the frontal and temporal lobes, especially in the basal areas [3]. Diffuse axonal injury is often the cause of the worst functional outcomes [2]. In this context, both motor and cognitive neurorehabilitation for TBI patients is fundamental to its beneficial and effective role in improving patient outcomes and quality of life [4].

Physiotherapy treatments are focused on recovering and/or improving balance and gait ability, activating the locomotor centres of the central nervous system (CNS) and, at the same time, strengthening the postural control needed for deambulation [5]. However, conventional rehabilitation techniques have some limits that may undermine the outcomes, including the absence of a standardised training environment, adaptable supports to more functionally train the patients as well as the ability to increase therapy intensity and dose with a reduced physical burden for the therapists [6]. Indeed, for example, physiotherapists have difficulties in ensuring spatial and temporal symmetry between the steps for severely affected patients, making the repeatability of the exercises imprecise [7]. New approaches for verticalization and gait training have been employed to overcome these problems, which are based on high-intensity training with a high number of task-oriented repetitions. This is possible thanks to robotic devices that can improve the reproducibility of kinematics during the gait cycle and increase the intensity and volume of motor exercises [7].

In the same way, devices for upper limb rehabilitation have been developed to guarantee the intensity and repeatability of shoulder, arm and hand movements [8], although the reproducibility of the upper limb kinematics is more complex. In particular, no single device has proven feasible and effective in rehabilitating all movements of the upper limb joints.

Moreover, cognitive and behavioural abnormalities following TBI represent the main problem in clinical practice. To better deal with it, cognitive rehabilitation is also evolving towards an innovative approach using virtual reality (VR) in order to not use only pencil/paper and other conventional tools. VR consists of an interactive and virtual environment in which patients can interact with computer-generated graphics and different degrees of immersive sensations [9]. Notably, thanks to the VR’s playful settings, the patient’s compliance increases by amplifying the effects of the rehabilitation treatment itself, which can also be customised according to the actual needs of the subject [10].

In addition, in patients with severe TBI (sTBI), walking training and specific cognitive programs make rehabilitation difficult or even impossible due to a lack of physical and psychiatric patient compliance [11]. These patients, especially when affected by disorder consciousness, could benefit from early robotic verticalization through tilt tables to avoid episodes of orthostatic hypotension and bedridden complications [12]. This is why robotics and VR may be a complementary treatment to further improve functional recovery in TBI. In fact, innovative neurorehabilitation tools could further stimulate spontaneous neuroplastic processes (such as angiogenesis, neurogenesis, and changes in cortical paths), subtending gait, upper limb and cognitive recovery [13]. Indeed, the spontaneous neuroplastic mechanisms are short-lived and sometimes too weak to counteract brain damage and avoid maladaptive plasticity [13,14,15]. For this reason, implementing neurorehabilitation interventions would be useful to stimulate specific sensory-motor and cognitive neuronal circuits, considering the level of injury and disability.

Nevertheless, robot technology for rehabilitation requires high levels of investment, especially for its maintenance and routine operation, compared to conventional interventions. This is why hospitals might be reluctant to adopt innovative technology in the rehabilitation field, also representing a barrier to their implementation [16]. However, a positive cost-effectiveness ratio on its use has been found in the last few years [17,18].

Although some factors may negatively affect the use of innovative technology in clinical practice, growing evidence is demonstrating that patients with neurological disorders may benefit from robotics, virtual reality and other tools [19,20]. In particular, following stroke, people who receive electromechanical-assisted gait training in combination with physiotherapy are more likely to achieve independent walking than people who receive conventional gait training alone [21].

Taking into consideration these positive effects in other diseases, the aim of this review was to systematically investigate the existing evidence concerning the role and the effects of technologies in motor and cognitive neurorehabilitation in patients with moderate to severe TBI.

## 2. Methods

### 2.1. Search Strategy

The systematic review was performed using the PRISMA (Preferred Reporting Items for Systematic Reviews and Meta-Analyses) guidelines [22] to investigate the role and the effects of technologies in motor and cognitive neurorehabilitation in patients with moderate to severe TBI. The data were collected using the following databases: Cochrane Library, PEDro, PubMed and Google Scholar, and the following keywords: “robotic rehabilitation” and/or “virtual reality rehabilitation” and/or “innovative approach” and/or “end effector”; “exoskeleton” and/or “neurorehabilitation” and/or “innovative cognitive rehabilitation”, all combined with the expression “traumatic brain injury”. Moreover, we have also analysed the references of the selected articles in order to obtain a complete search.

### 2.2. PICO Evaluation

We defined our combination of search terms using a PICO (population, intervention, comparison, outcome) model. The population was limited to moderate to severe TBI patients; intervention included all the innovative rehabilitative approaches, including robotic devices, virtual reality and computer-based training, telerehabilitation, neuromodulation; the comparison was evaluated considering the standard cognitive and motor rehabilitation techniques; and outcome included any motor and cognitive improvements shown by the patients, efficacy of treatment.

### 2.3. Inclusion and Exclusion Criteria

The inclusion criteria were (i) patients affected by moderate to severe TBI; (ii) randomised clinical trials (RCT), pilot studies and systematic reviews published between January 2012 and September 2022; (iii) English language; and (iv) published in a peer-reviewed journal. Exclusion criteria were (i) case reports, case control and retrospective studies, and narrative reviews; (ii) studies with other kinds of electromedical devices; (iii) studies involving mild TBI, children and adolescents; (iv) other pathologies (i.e., vascular accidents, ischaemic and/or hemorrhagic).

### 2.4. Literature Selection

**Figure 1:**
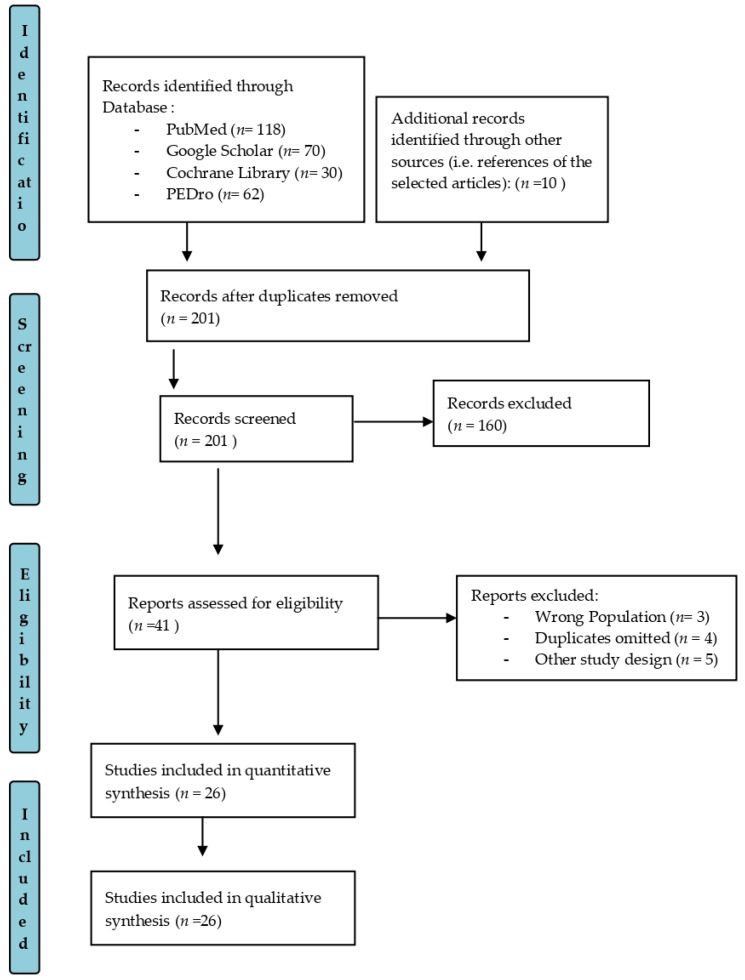
PRISMA flow diagram for study selection.

Two reviewers (MB and RDL) screened for relevance to the motor and cognitive effects of innovative rehabilitative approaches. Then, abstracts of the remaining articles were read, and those not meeting the eligibility criteria were excluded. The full text of all potential articles was evaluated in depth. In case of uncertainty, or when the abstract was not available, the entire article was read. If a disagreement was present, a third reviewer solved the concern (RSC). A PRISMA flow diagram was used for study selection (Figure 1). Since this was intended only as a comprehensive review, we did not perform a meta-analysis of the data. Of the 290 screened studies, 41 were selected, and eventually 26 were included in this review as they met the inclusion criteria (Figure 1).

### 2.5. Study Quality Assessment

In addition, three authors rated the studies included in this review, using the Physiotherapy Evidence Database (PEDro) scoring system [23] to assess the methodological quality of studies. PEDro assesses 11 areas of study quality that are answered with a “yes” (score = 1) or “no” (score = 0). The first item is a measure of external validity and is not used in calculating the final score (i.e., sum of items 2–11).

## 3. Results

Twenty-six articles were eventually analysed as they met our inclusion criteria. Given the different devices we dealt with, the results have been divided into six sections: Robotic and Virtual Systems for Motor Rehabilitation (*n* = 8); Humanoid Robots (*n* = 1); Virtual Reality Systems for Cognitive rehabilitation (*n* = 7); Computer-based rehabilitative approach (*n* = 2); Tele-rehabilitation (*n* = 3); Neuromodulation and combined approaches (*n* = 5).

The PEDro analysis revealed that among the selected studies, four studies presented a score of 6, one study with a score of 7, two studies with a score of 5, while the others ranged between 4–2.

Most of the included articles, independently of the device used, presented a higher prevalence of cognitive than motor outcomes (Table 1).

### 3.1. Robotic and Virtual Systems for Motor Rehabilitation

Among the 26 articles included in this review, we found only 2 articles [12,24] on the use of robotic verticalization with the Erigo device in sTBI patients. Specifically, De Luca et al. [12] administered autobiographical music stimulation plus robotic verticalization to the experimental group with a better improvement in the level of awareness and consciousness. Taveggia et al. [24] found that the use of the Erigo device may reduce orthostatic hypotension in vs. and MCS patients, thanks to its gradual verticalization and stepping, with constant and non-invasive monitoring of vitals parameters.

Three studies [25,26,27] investigated the use of RAGT by means of the Lokomat device, a robotic orthosis that guides the hip and the knee through a treadmill on which the patient’s body weight may be supported/reduced. In detail, one study [25] found a 21%, 45% and 38% increase in patients’ self-selected walking velocity when comparing the G-EO system, Lokomat and partial-body weight-supported treadmill training, respectively. In addition, the Lokomat and G-EO groups presented a more symmetrical gait pattern due to the advancement of the paretic limb. Williams et al. [26] suggested that RAGT may be a safe and feasible intervention that can be used as part of a physical therapy plan of care in adults with DoC due to TBI. Another study [27] compared the effects of the Lokomat with manually assisted treadmill training (MATT) in 16 TBI patients with limitations in ambulation. Beyond a significant improvement in step-length symmetry, the Lokomat required fewer staff and less manual effort with decreased staffing costs.

Only three studies [28,29,30] included the use of VR in the achievement of motor outcomes. One randomised controlled trial [28] used balance-based physical therapy by means of a Nintendo Wii in addition to their standard physical therapy regimen for improving static balance. The advanced training was personalised to the patient’s specific needs and included different surfaces, bases of support, visual inputs, as well as dual-task and dynamic interactive activities. Postural treatment using VR (i.e., virtual games with Kinect Motion sensor) was investigated by a pilot study [29] which trained and improved limb coordination, posture and gait after 15 training sessions. Another study [30], comparing VR training using the Xbox Kinect games with a home-based exercise program, found that both groups improved in balance without any statistical differences.

### 3.2. Humanoid Robots

We found only one study [31] on the use of Humanoid robots, known as social robots, in the treatment of cognitive functions and social skills in TBI. The authors [31] showed the positive effects of the human robot Pepper on the improvement of cognitive and emotive processes, communication, and social skills compared to traditional cognitive treatment.

### 3.3. Virtual Reality Systems for Cognitive Rehabilitation

Five clinical studies [32,33,36,37] and two systematic reviews [34,35] on the cognitive effects of VR systems in TBI Neurorehabilitation were included. In particular, VR was used in two pilot studies [32,33] for the training of attention processes: a visuo-haptic virtual environment and a virtual touch modality by means of the Virtual Reality Rehabilitation System (VRRS) were used to improve cognitive function. The two systematic reviews investigated VR gaming for cognitive treatment of moderate TBI, demonstrating that VR tools may improve memory and executive function in patients with TBI, while for attention training, weak evidence still exists [34,35]. A pilot study [36] carried out in TBI military patients tested the effect of NeuroDRIVE on driving abilities, cognitive performance and neurobehavioral symptoms, and demonstrated statistically-significant improvements in working memory and visual search/selective attention. Another pilot study [37] considered a virtual supermarket environment, namely the VMall, to perform occupational therapy compared to non-VR occupational therapy cognitive retraining. The authors suggested that cognitive treatment that focuses on mediating strategies can improve executive functions and also IADL independently of the use of VR environments. Finally, the BTs-Nirvana non-immersive VR system was used in a clinical trial with one hundred TBI patients [38], showing a great improvement in specific cognitive domains, such as cognitive flexibility, attentional shifting, visual search, and executive and visuospatial functions that are necessary for planning and managing daily life.

### 3.4. Computer-Based Rehabilitative Approach

We found only two papers [39,40] about the use of computer-based interventions to improve cognitive functioning in subjects following TBI. Lebowitz and colleagues [39] evaluated the feasibility and utility of computer-based mental exercises for visual processing and memory, i.e., the Cortex with InSight (Posit Science Corporation, San Francisco, California), for individuals with a history of TBI at home. This advanced training optimised the main cognitive functions, including attention, memory and information processing. Zickefoose et al. [40] examined the potential effect of two computerised brain game software programs in participants with severe TBI with a mean time post-injury of 4 years. Two participants received either four weeks of computer-based Attention Process Training (APT-3) first or Lumosity brain games first and were observed for 30 min per day, five days per week. Then, they switched to the other intervention for another four weeks. All participants significantly improved their level of difficulty on intervention tasks, and there was a trend toward generalisation to daily tasks.

### 3.5. Tele-Rehabilitation

We found one systematic review [41] and two studies [42,43] on the use of information and communication technologies, including telephone, messaging, e-mail, and video conferencing systems, in the rehabilitation of TBI. Ownsworth et al. [41] evaluated the efficacy of telerehabilitation through telephone-based (10 studies) and Internet-based (3 studies) interventions for adults with TBI, showing that the structured telephone interventions were effective in improving mood symptoms, functional status, emotional well-being, QoL and sleep quality. De Luca et al. [42], in their feasibility/usability study in severe TBI patients, found that the Virtual Reality Rehabilitation System (VRRS) had a beneficial effect on the patient’s motivation during the training, encouraging the return home and the continuity of care in the territory. Raso et al. [43] confirmed the potential use of telehealth for the management of DoC due to TBI through an advanced video conferencing telehealth system at home.

### 3.6. Neuromodulation and Combined Approaches

In one pilot study, the authors [44] suggested a potential role of neuromodulation, when applied to the left dorsolateral prefrontal cortex, in improving attention during cognitive training after TBI. The systematic review [45] involved five articles, two about repetitive transcranial magnetic stimulation (rTMS) and three on transcranial direct current stimulation (tDCS), suggesting that neural plasticity changes induced by Non-invasive brain stimulation (NIBS) may contribute to greater improvements when combined with rehabilitation. Another study [46] used NIBS to improve divided attention and to boost neural plasticity with a normalisation of the abnormal hyperactivations of the temporal, middle frontal and postcentral gyrus. These results were confirmed and implemented by Ulam et al. [47], suggesting that 10 anodal tDCS sessions may positively modulate cortical excitability in patients with TBI. On the contrary, Leśniak et al. provided insufficient evidence to support the efficacy of repeated a-tDCS for enhancing the rehabilitation of memory and attention in patients after severe TBI [48].

## 4. Discussion

To the best of our knowledge, this is the first systematic review that investigates the use of robotics and other technologies in moderate to severe TBI rehabilitation. Indeed, most existing reviews are focused only/mainly on VR approaches and cognitive telerehabilitation [33,34,40] and do not offer an overview of the most innovative approaches for both motor and cognitive rehabilitation in this patient population.

In Table 2, we have summarised the main innovative neurorehabilitation tools that may positively affect outcomes in TBI, providing information on their structure, function and use in the different stages/degrees of the disease.

Recently, the interest in the development of rehabilitative robotic systems has grown strongly. In fact, traditional approaches can improve functional recovery but require a considerable commitment of human resources, and it can be very challenging in patients with severe disabilities. Therefore, robotic devices represent valid support, as they allow repetitive, intensive and more lasting therapy, also guaranteeing a more objective assessment of the patients’ damage and outcomes. In particular, robotic motor systems allow a very early verticalization, as well as walking training, once the patients’ vital signs are stable. In fact, some devices, like the Lokomat, are capable of assisting a passive gait, so they can also be used in sTBI [49]. Afterwards, the gradual recovery of muscle strength will allow the therapist to reduce the BWS with the robotic device [50]. In fact, these robotic motor devices guarantee better postural and trunk control, decreasing muscle stiffness and acting on the improvement of cardio-circulatory and respiratory functions [51]. Noteworthy, available Clinical Practice Guidelines to improve locomotor function following brain injury [52] indicate that clinicians should offer walking training at moderate to high intensities or virtual reality-based training to ambulatory individuals greater than six months following the brain injury acute-onset to improve walking speed or distance. Indeed, Esquenazi et al. [25] showed that an intensive RAGT with G-EO system or Lokomat in chronic TBI patients can improve velocity, endurance and symmetry and lengths of steps. Otherwise, weak evidence suggests that strength training, circuit (i.e., combined) training or cycling training at moderate to high intensities, and virtual reality-based balance training may improve walking speed and distance in these patient groups. However, the optimal therapeutic intervention to achieve full gait recovery remains unknown, but it is evident that any rehabilitative effort to guide motor recovery should encounter fundamentals of neuroplasticity and be related to clinical prognostic indicators (i.e., disease severity, vital signs, intracranial pressure, cerebral perfusion pressure) [53,54].

**Table 2:** Description of the main innovative neurorehabilitation tool used in TBI patients.

Robotic Device	Short Description	Picture
Erigo [12,24]	Erigo is a robotic tilt table used in the early stage of recovery after an acute TBI. It allows an early and gradual robotic verticalization combined with a cyclic leg movement in order to stimulate CNS through critical afferent stimuli. The tilt table inclination can be regulated by the therapists, who can control the gradualness of verticalization (i.e., from 45° to 90°) as well as the stepping speed, according to the patients’ needs. In addition, the Erigo device also can improve cardiocirculatory stability through muscle activation, pump function and venous return.	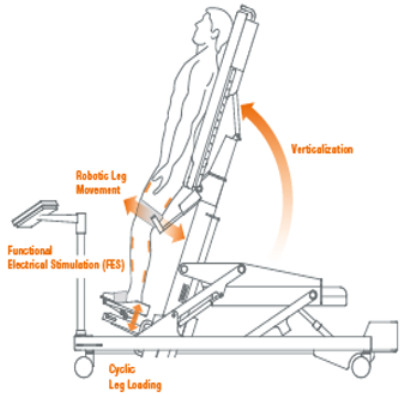
Lokomat [25,26,27]	The Lokomat is a robotic gait-assisted device widely used in the neurorehabilitation of walking. It consists of an external gait orthosis integrated with a computer-controlled linear actuators at each hip and knee joint, in addition to an advanced system of body weight support system and a treadmill. The therapists can control the level of gait assisted-support, the force and the pattern of gait movement.	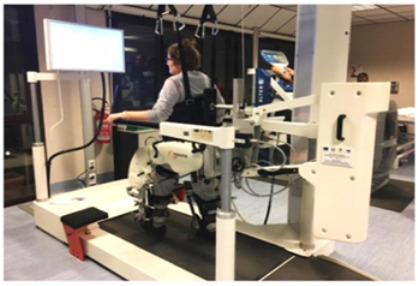
GEO-O [25]	GEO SYSTEM is a robotic end effector system that simulates the repetitive training of locomotion in everyday life, such as walking on the flat, ascending and descending stairs. The patient’s feet are secured to the platforms moving in all directions (i.e., upwards, downwards, forwards and backwards) with the assistance of six engines. There is also an Evolution version that also includes the use of an immersive scenario to fully involve the person in rehabilitation therapy in dual-task activities.	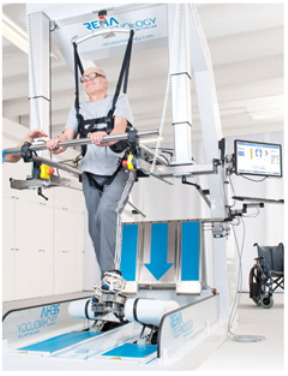
**Human-Robot**	**Short Description**	**Picture**
Robot-PEPPER [31]	Pepper is one of the most widely known social humanoid robots, which is used to recognize faces and basic human emotions. This humanoid robot was conceptualised for human interaction, thanks to the conversation and his colourful touchscreen.	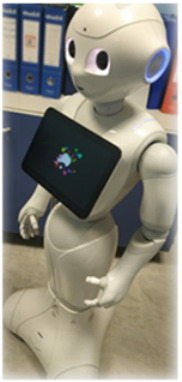
Robot-NAO [55]	Nao is another humanoid robot characterised by its small size, and it is able to interact with adult, adolescent and paediatric patients. It is equipped with sensors that allow it to walk, dance, speak, and recognize faces and objects. It can be used to provide social activities and to recognize emotions, giving sensorial feedback to patients.	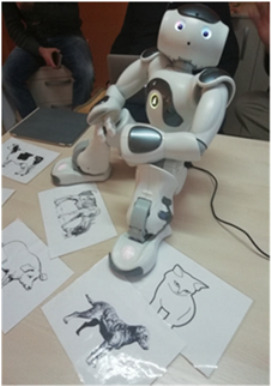
**Virtual Reality System**	**Short Description**	**Picture**
BTS Nirvana [38]	BTS NIRVANA is an innovative therapeutic system that assists the rehabilitation process of patients affected by neurological diseases, thanks to its multi-sensorial stimulation. The patients can move or manipulate specific objects in different ways (i.e., balls, flowers, and butterflies) or create specific combinations (i.e., colour-number) with a dynamic involvement in the virtual environment. During the interaction between the patient and the screen, the system produces audio and video feedback (using the sprite activity). The difficulty of exercises increases the base of the number of distractors and reduces the time available for the execution.	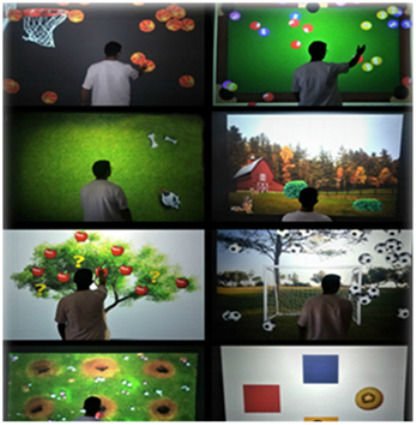
VRRS Virtual Reality Rehabilitation System [33]	Il Khymeia VRRS—Virtual Reality Rehabilitation System—is the most widely used for VR training and teletraining in clinical practice. The VRRS, in fact, is conceptualised with a “central HUB” that can be connected via USB, a series of specialised peripherals fully synchronised and integrated with the system. The VRRS is equipped with exercise modules for cognitive, language, postural, and motor rehabilitation. These virtual exercises can be selected and included in the rehab program by the therapist, who can shape the difficulty in relation to the time of execution and the type of activity.	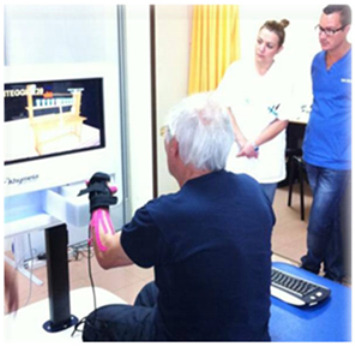
Neuro-DRIVE System [36]	The Neuro-DRIVE system uses a virtual reality driving simulator. It consists of a curved screen in addition to a driving console similar to a typical automobile. Each driving console presents turn signals, gas and brake pedals, a steering wheel, a digital dashboard, and a seat belt. The patient is seated in front of the screen, holding the steering wheel and pushing the pedals while interacting with the virtual environment stimuli.	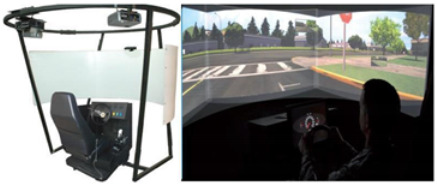

People with TBI are affected by cognitive-behavioural problems, and many devices have been developed and used in research and clinical practice to deal with these problems. Concerning human-like robots, also called Socially Assistive Robots (SARs), such as PEPPER and Nao, primarily assist people in social interactions (e.g., speaking, driving, remembering, observing, and entertaining). In TBI patients, SARs [31] enhance mood, social relationships among patients, and emotional expression of brain injury survivors. Assad-Uz-Zaman [55] implemented the software-library exercise programs of Nao robots to be administered in individuals with upper limb impairment and also in the TBI population.

VR systems, indeed, are considered one of the most innovative technologies to potentiate both motor and cognitive rehabilitation. In fact, it is evident that by using this tool, several very intense types of exercise can be adapted to achieve specific outcomes for TBI patients. In fact, VR can be used to provide the patient with repetitive, task-oriented training due to the multisensorial feedback, thus potentiating the use-dependent plasticity of the sensory-motor cortex [56,57,58]. Patients interacting with a virtual environment could be trained in a more ecological way and can perform all those activities that are dangerous to be performed in real life. VR, by providing individuals with “knowledge of results” and “knowledge of performance” may favour reinforcement learning and then functional recovery. Nonetheless, our systematic analysis showed poor findings supporting VR for balance training using Nintendo Wii and Xbox Kinect sensors [29,30], and the VR training was no more effective than the standard treatment. Some specific immersive VR devices, such as the Computer-Assisted Rehabilitation Environment (CAREN), may lead to better results. The tool is able to provide the patient with a sense of presence, acting through both bottom-up (i.e., the neurosensorial feedback given by the treadmill and the six degrees of freedom balance) and top-down (VR acoustic and visual) modalities. Then, it has been successfully used to improve motor and cognitive abilities, vestibular dysfunction, as well as mood and anxiety disorders, especially in military medicine [59,60,61].

Computer-based treatment (C-BT) has been used for cognitive training in patients with moderate to severe TBI, and some authors have demonstrated that enjoyment and active involvement may lead to better results [40]. A critical review of the literature suggested that C-BT seems promising as an approach to improve cognition, with regard to working memory, in TBI patients [62]. Notably, it has been hypothesised that attention improvements with this tool are related to a combination of factors, such as young age, years since the trauma, closed head injury, higher pre-intervention scores on the Test of Everyday Attention, and this should be taken into account when dealing with C-BT.

Another growing issue concerns the use of telemedicine and ehealth in the rehabilitation field [63,64], although, in our review, we found only a few articles dealing with TR in the TBI population. Despite the poor available data, it is suggested that TR could be of help by overcoming the displacement of therapists or patients and reduction of patient hospitalisation times and costs for both patients and healthcare providers [65]. In this way, telehealth systems allow cost-effectiveness avoiding feelings of abandonment and stress from both patients and caregivers [66].

Finally, NIBS is demonstrating a positive effect in promoting neuroplasticity and functional recovery following a brain injury [45], thus further potentiating cognitive rehabilitation effects [44]. Better and long-lasting results are achieved when neuromodulation is applied in combination with other methodologies, including computer-assisted training [46]. Moreover, among the potential sites of stimulation, the dorsolateral prefrontal cortex (DLPFC) seems to be preferential for its association with attention and working memory function [46,48], although it is under debate which kind of current should be used (cathodal vs. anodal) and the time/frequency of the stimulation protocol. However, it seems that the combined application of anodal and cathodal current on the right DLPFC produces improvement in cognitive performance [67].

To summarise, since various innovative neurorehabilitation approaches exist, clinicians, as well as researchers, do not always agree on which technology is better to train TBI patients. The approach’s choice should take into account the site and the extent of brain damage, the age and the severity of the clinical status (i.e., a disorder of consciousness, severe vs. moderate motor and cognitive disability) [68]. In addition, patients affected by TBI (or at least their caregivers) should be familiar with technical issues to not yield the training more challenging [42], especially when they have to use virtual reality, computer-based approaches and telerehabilitation. In detail:

(1) Patients with a diagnosis of DoC or sTBI need to achieve an early and gradual verticalization in order to avoid deterioration of the autonomic nervous system and bedridden complications [12,24]. For these reasons, the Erigo device could be a useful tool in clinical practice to meet patient necessities. In addition, sTBI could benefit from RAGT through Lokomat, which can assist a passive gait-increasing BWS, always monitoring vital parameters to guarantee a safe and feasible rehabilitative intervention [26].
(2) Patients affected by moderate TBI may gain balance and coordination thanks to VR exercises, which are also known to promote the enjoyment and active involvement of the patients [28,29,30], even if the evidence found is not sufficient to support its systematic use in TBI clinical practice. Instead, the GE-O system seems to be a valid tool for gait training in moderate TBI patients to improve endurance and walking speed [25].
(3) On the other hand, the C-BT, as well as VR systems, are widely used in severe and moderate TBI patients to train cognitive functions. VRRS allows a specific subdomain training and, thanks to its big screen, provides a wider view of space and facilitates the execution of cognitive tasks [33,38], whereas moderate TBI could take advantage of C-BT, which requires more control in the upper limb [39,40].
(4) Another useful innovative approach is TR; despite the poor evidence in the TBI population, it can be useful in ensuring continuity of care between discharge from the hospital and return to home [42], avoiding travelling costs and geographical barriers for patients living far from metropolitan areas [65,66]. Specifically, the VRRS for TR is a feasible tool for moderate TBI patients that allows therapists to plan a specific training session (i.e., motor, with regard to upper limb, and cognitive) remotely supervised by the Tele-CockPit workstation [42].
(5) Last, NIBS in the moderate to severe TBI population is a promising approach, especially used to stimulate cognitive functions [67]. However, it is still an emerging approach and there is not enough data to confirm its use in clinical practice. Possible future directions could investigate the efficacy of NIBS combined with other technologies such as TR, C-BT and VR.

This systematic review has some limitations to acknowledge, including a lack of bias analysis, the wide variability of available technologies and an absent meta-analysis of the data. The selected studies also presented limitations, including small sample sizes, lack of control group and lack of long-term follow-up evaluations. We have instead comprehensively considered all the technologies and their effects used in the motor and cognitive neurorehabilitation of moderate to severe TBI patients.

## 5. Conclusions

This review provides clinically useful evidence on the safety and potential efficacy of technologies in TBI in an attempt to bridge the gap between research and clinical practice in this frail patient population. Our results revealed that the use of innovative technologies in TBI neurorehabilitation provided improvements in motor and, above all, cognitive outcomes. Although evidence on the effectiveness of advanced motor rehabilitation is still poor, it seems to be promising, at least in some sTBI. For these reasons, further and larger studies are needed to identify whether and to what extent patients with sTBI may benefit from robotic devices, VR and neuromodulation.

## Figures and Tables

**Table 1:** Robotics and technologies used in TBI neurorehabilitation.

Reference	Sample Size (Subjects Affected)	Study Design	Severity	Robotic Device/Advanced Approach	Outcome Measure	Intensity of Duration Training	Major Findings	PEDro Score
[12] (De Luca et al., 2022)	16	Pilot study	MCS (9 plus and 7 minus)	Erigo device for verticalization plus music stimulation	CRS-r; LCF; FIM; FCS; TCT;	Three times a week, for about 8 consecutive weeks, each session lasting about 45 min	In the experimental group, significant changes were found in patients’ awareness, global functional outcome, and non-verbal skills, while the control group, showed improvements in the individual scores without reaching a statistical significance.	6
[24] (Taveggia et al., 2015)	8	RCT	VS and MCS	Erigo device with a constant monitoring of Blood pressure and rate with SOMNOscreen plus, a polygraphy device	LCF, CRS-r; blood pressure; heart rate	Each training was performed in the morning in appropriate physical therapy room. Sessions were repeated three times a week for 24 sessions.	Training with the Erigo device, thanks to the integrated cycling movements, decreased the cardiovascular distress in MCS and VS patients with hemodynamic instability.	3
[25] (Esquenazi et al., 2017)	22	Randomised, prospective, pilot study	Moderate TBI	G-EO; Lokomat; manual assisted BWSTT	SSV; MV; spatiotemporal asymmetry ratio; 6MWT; MSIS	The authors administered 18 sessions of gait training for 6 to 8 weeks, generally 3 times per week. Each session lasted up to 75 min	Gait training rehabilitation using G-EO, Lokomat, or PBWSTT in individuals with chronic TBI improved SSV. MV was improved only in Lokomat and BWSTT groups; patients treated with G-EO and PBWSTT significantly increased their endurance (6MWT) in the post-training	6
[26] (Williams et al., 2020)	4	Clinical Trial	MCS (lack of functional communication or ability to consistently follow commands)	RAGT with Lokomat	HR; MAP; SaO2; ABS; FLACC; CRS-r; MAS; RLA	5 to 20 min, over 1- to 2-week periods.	Authors suggested that RAGT could be used in DoC due to TBI, as a safe and feasible intervention, as a part of a physical therapy program in adults with DoC due to TBI. However, they reported that two patients had experienced adverse symptoms such as pain from harness placement and increased somnolence.	1
[27] (Esquenazi et al., 2013)	16	Randomised prospective study	Moderate TBI	RATT and MATT	SSV;MV; spatiotemporal asymmetry ratio; 6MWT; MSIS	Each training lasted 45 min, 3 times a week with either RATT or MATT for a total of 18 training sessions.	Both patient groups showed significant improvement in their motor function. However, no training technique appears to be superior to the other, between the two intervention groups.	4
[28] (Cuthbert et al., 2014)	20	Randomised controlled trial	Moderate TBI	Nintendo Wii (virtual reality gaming system)	PACES; BBS; FGA	8 min of Wii Fit balance board games and 7 min of Wii Sport games, 4-times per week	The authors presented that the administration of Wii balance board is more useful to informally assess or treat static balance alteration rather than for dynamic balance.	2
[29] (Ustinova et al., 2014)	15	Pilot study	Moderate TBI	interactive, customised VR games and scenarios, utilising an Xbox Kinect sensor	BBS; FGA; KAS; FRT	Patients underwent 15 sessions, lasting 50–55 min, scheduled 2–3 times a week over 5–6 consecutive week	This study showed that patients affected by chronic impairments due to TBI increased their postural stability, gait, arm movement and coordination, thanks to VR training.	4
[30] (Teterfiller et al., 2019)	63	Randomised Controlled Trial	Moderate to severe TBI	Xbox Kinect games	CB&MS; BESTest; ABC; PART-O	Each session was scheduled 3–4 times per week for 12 weeks, lasting for 30 min.	No statistical significance between the two groups was achieved; however, both treatment groups presented better balance responses to these interventions in chronic TBI patients.	6
[31] (Corallo et al., 2022)	12	Pilot study	Severe TBI	Human-Robot Pepper	LCF; MMSE; SIB; BDI I-II; HAM-A; FIM; EQ-5D	Each session was scheduled for 3 times a week for 8 weeks, lasting for 60 min.	Results demonstrated that the experimental group presented better improvements in mood symptoms and in QoL than controls, unlike their cognitive performance that did not register any improvement.	5
[32] (Dvorkin et al., 2013)	21	Pilot study	Severe TBI	VRROOM system (Virtual Reality and Robotics Optical Operations Machine)	Kinematic analysis of arm movements; RLA	Patients were evaluated for two consecutive days about 3 conditions (no haptic feedback, a break-through force, and haptic nudge) lasting for 12 successive, 4-min blocks.	Patients well-tolerated the Visuo-haptic environments that promoted motor functions in TBI population.	4
[33] (De Luca et al., 2022)	30	Pilot study	Severe TBI	VRRS	MoCA; AM; TMT; HRS-D	Training session was organised for 3 times a week for 8 weeks (i.e., 24 sessions lasting for 45 min each).	The authors found great improvements in cognitive performance and mood symptoms in both two groups, especially in the experimental group, which received training using the VRRS; improvements were registered in each specific attention (i.e., visual attention, task switching, visual search speed, etc.).	5
[34] (Alasharam et al., 2019)	9 studies	Systematic review	Moderate to Severe	Semi-Immersive and non-immersive virtual systems	Memory (Digit Span); Executive Functioning (WSCT and London Tower); Attention	Ten sessions, 3–4 times per week, 45 min, 2 times per week for 6 weeks, 30–45 min in duration, received 12 sessions of 20–25 min, received 8 sessions, 2 times per week for 4 weeks, 60 min in duration	This systematic review concluded that VR training intervention may improve memory and executive function as an aspect of cognitive function in patients with TBI. However, weak evidence was found about the positive effect of VR intervention on attention post-TBI.	*n*/A
[35] (Ausilio et al., 2020)	12 studies	Systematic review	Moderate to severe	CAREN, VROOM, Kinect sensors, VMall	Gait; Balance; Cognition	Two times—6 weeks, 2 times—2 months, 10 sessions, 12 sessions, 12 sessions over 4 weeks	VR training has been shown to have a potentially beneficial role in gait and balance deficits in patients with TBI. However, the evidence about the use of VR systems in the treatment of upper limb functioning is still limited in comparison. Finally, the use of VR for cognitive rehabilitation is widely supported, especially in TBI patients.	*n*/A
[36] (Ettenhofer et al., 2019)	11	Pilot study	Moderate to severe	NeuroDRIVE	VR Driving Assessment; TMT; WAIS-IV; COWATLA; CVLT-II; Grooved Pegboard; NSI; BDI; Epworth Sleepiness Scale; FSS; SF-36; Satisfaction with Life Scale	Six 90-min sessions (9 h total) conducted over a four-week period	The NeuroDRIVE intervention could be a valuable and useful tool to train cognitive functioning; although it seems that this innovative intervention does not produce improvements in driving abilities, measured with the VR driving assessment.	2
[37] (Jacoby et al., 2013)	12	Pilot study	Moderate to severe	VMall, virtual supermarket environment— is operated via GestureTek’s Interactive Rehabilitation and Exercise System (IREX) video capture system	EFPT; MET-SV	Ten 45-min treatment sessions, 3–4 times per week via the VMall environment	Both kinds of treatment, through VR and not, showed improvements in the IADL activities. Standard OT (without VR) showed better outcomes in daily activities.	3
[38] (De Luca et al. 2019)	100	Clinical trial	Moderate TBI	BTS-Nirvana	MoCA; HRS-D; HRS-A; TMT; VS; FAB; WEIGL	Total of 24 1 h sessions (3 times a week for 8 weeks)	Each treatment produced improvements in cognitive functioning in addition to mood. However, the authors found that only the experimental group presented significantly better results in cognitive flexibility and shifting skills (TMT B-A) and in selective attention/visual research (VS).	4
[39] (Lebowitz et al., 2012)	10	Pilot study	Moderate to severe	Cortex with InSight	ANAM4, a validated, computerized neuropsychological battery that takes about 30 min to administer; CFQ; FrSBe	At home 40 min/d, 5 d/wk for 6 wk	The authors concluded that the use of PC software could be a promising and well-tolerated therapy for TBI patients. Only fatigue was reported by the participants, as a side effect.	2
[40] (Zickefoose et al. 2013)	4	Pilot study	Severe TBI	Attention Process Training-3 (APT-3) and Lumosity™ (2010) Brain Games	WAB-r; NAB, and a designed measure to inform about patients’ perceptions regarding the two intervention programmes	Twenty treatment sessions within a 1-month period, with each session lasting 30 min.	The four patients showed progressive improvements in reaching new levels of difficulty on the tasks during the APT-3 and Lumosity training.	1
[41] (Ownsworth et al., 2018)	13 studies	Systematic review	Moderate to severe	Telerehabilitation about the feasibility and/or efficacy of telephone-based (10 studies) and Internet-based (3 studies) interventions	Functional status and depressive symptoms, global composite of functioning, Self-report measures assessed mood, behaviour. Family caregivers reported everyday memory problems, goals, and strategy use	Eight-week in-hospital cognitive rehabilitation program, the 8 × 30-min telephone counselling sessions	Telephone-based interventions were shown to be a promising tool to allow access to specific remote interventions for people with TBI. However, this intervention was limited to short-term outcomes. On the other hand, internet-based intervention studies were focused on the feasibility of web systems.	*n*/A
[42] (De Luca et al., 2020)	10	Feasibility and usability study	Severe TBI	Telerehabilitation System VRRS	IMI and SUS	Six training sessions, provided 3 times per week for two weeks, each session lasting about one hour	The telerehabilitation system showed to have good usability, in addition to its advantages, such as facilitating hospital discharge, and optimising motivation during the training.	1
[43] (Raso et al., 2021)	22	Pilot study	VS and MCS	Advanced video conferencing telehealth system for controlling neurological patients at home.	CSR-r, LCF, WHIM, NCS.	24 h/d, 7 d/wk for monitoring all basic care activities.	Telehealthcare system demonstrated to be not inferior to usual in-person care, to manage DoC due to TBI.	7
[44] (Kang et al., 2012)	9	Double-blinded pilot study	Moderate to Severe	Anodal transcranial direct current stimulation	Computerised contrast reaction time task and numeric rating scale describing levels of attention, fatigue, task difficulty, and sleep quality	tDCS stimulation was delivered for 20 min	The authors demonstrated that the application of an excitatory anodal tDCS on the left DLPFC was associated with shortened reaction times in TBI patients, while the sham stimulation had no discernible effects. This study concluded that NIBS could be used to stimulate attention in TBI people.	6
[45] (Hara et al., 2021)	5 studies	Systematic review	Moderate to severe	rTMS and tDCS	rTMS, target symptoms included attention (*n* = 2), memory (*n* = 1), and executive function (*n* = 2) tDCS studies, target symptoms included cognition (*n* = 2), attention (*n* = 3), memory (*n* = 3), working memory (*n* = 3), and executive function (*n* = 1)	10 Hz 110% MT 2000 pulses/session, 1 Hz 100% MT 2000 pulses/session, 2 mA/35 cm^2^ × 20 min. Two anodes, 1 mA/25 cm^2^ × 20 min, Anodal electrode, 1 mA/10 min/current density = 0.028 mA/cm^2^, Anodal tDCS	In this systematic review, DLPFC was chosen as the preferential stimulation site in all included studies. In some cases, authors registered improvements in comparison with the control group. However, which method is more effective between rTMS or tDCS remains unknown. In conclusion, the authors pointed out that NIBS is more likely to produce improvements when it is combined with other rehabilitative approaches.	*n*/A
[46] (Sacco et al., 2016)	32	Pilot study	Severe TBI	tDCS stimulation was performed using a HDCstim device (Newronika srl, Milan, Italy).	TEA; RBANS; BDI; AES	Ten sessions, each session included 20 min of tDCS stimulation followed by 30 min of cognitive training	The proposed treatment produced promising positive results in Divided Attention. In addition, the association between tDCS and computer-based training may have allowed a neural reorganisation, reducing the patients’ cognitive effort.	4
[47] (Ulam et al., 2015)	26	Randomised, double-blind study	Moderate to severe	Anodal tDCS on EEG oscillations	Digit Span; WAIS IV	20 min sessions of 1 mA anodal stimulation to the left dorsolateral prefrontal cortex (F3, cathode placed at right supraorbital site, Fp2) were provided on 10 consecutive days	In this study, the authors concluded that the use of tDCS can be an emerging tool for the treatment of the neuropsychological sequelae due to TBI, also in the subacute stage of recovery.	3
[48] (Lesniak et al., 2014)	23	Pilot randomised controlled trial	Severe TBI	Transcranial direct current stimulation (A-tDCS) of the left dorsolateral prefrontal cortex	RAVLT; PRM; PASAT; SSP; RVP; EBIQ	A-tDCS (10 min; 1 mA; in the DLPFC, followed by rehabilitative cognitive training daily for 15 days.	The use of tDCS increased the score of most outcome measures, including an auditory verbal memory test, 2 working memory tests, and an attention test, registering a positive response to stimulation, although there were no statistical differences between the experimental and control groups.	2

Legend: MCS (minimally conscious state), MCS plus (patients with specific pivotal behaviors, such as consistent and reproducible movement to command, object recognition and intelligible verbalization with intentional (nonfunctional) communication), MCS minus (patients with reaching, visual pursuit, fixation, object manipulation, and automatic motor response); CRS-r (Coma Recovery Scale-revised), LCF(Level of Cognitive Functioning), FIM (Functional Independence Measure), FCS (Functional Communication Scale), TCT (Trunk Control Test), RV (Robotic Verticalization), EFA (Early Functional ability), PBWSTT (partial-body weight–supported treadmill training), SSV (self-selected velocity), MV (maximal velocity), 6-MWT (6-Minute Walk Test), MSIS (mobility domain of Stroke Impact Scale), HR (Heart Rate), MAP (Mean Arterial Pressure), SaO2 (Oxygen Saturation), ABS (Agitation Behavior Scale), FLACC (Face, Legs, Activity, Cry, Consolability Scale), MAS (Modified Ashworth Scale), RLA (Rancho Los Amigos), RATT (robotic-assisted treadmill training), MATT (manually assisted treadmill training), PACES (Physical Activity Enjoyment Scale), BBS (Berg Balance Scale), FGA (Functional Gait Assessment), KAS (Klockgether Ataxia Scale), FRT (Functional Reach Test), CB&MS (Community Balance and Mobility Scale), BESTest (Balance Evaluation System Test), ABC (Activities-Specific Balance Confidence Scale), PART-O (Participation Assessment with Recombined Tools-Objective), MoCA (Montreal Cognitive Assessment), WEIGL (Weigl’s Sorting Test), FAB (Frontal Assessment Battery), vs. (Visual Search), TMT (Trail Making Test), BDI (Beck Depression Inventory), SF-12(Short Form-12), MMSE (Mini Mental State Examination), SIB (Severe Impairment Battery), HAM-A (Hamilton Rating Scale for anxiety), EQ-5D (EuroQol-5D), AM (Attention Matrices), HRS-D (Hamilton Rating Scale—Depression), NeuroDRIVE (Neurocognitive Driving Rehabilitation in Virtual Environments), COWATLA (Controlled Oral Word Association Test, Letters & Animals), CVLT-II (California Verbal Learning Test-II), NSI (Neurobehavioral Symptom Inventory), FSS (Fatigue Severity Scale), GOS-E(Glasgow Outcome Scale-Extended), MET-SV (Multiple Errands Test—Simplified Version), EFPT (Executive Function Performance Test),ANAM4(Automated Neuropsychological Assessment Metrics Version 4), CFQ (The Cognitive Failures Questionnaire), FrSBe (Frontal Systems Behaviour Scale), WAB-r (Western Aphasia Battery–Revised), NAB (Neurological Assessment Battery), TEA (Examination of Attention), RBANS (Repeatable Battery for the Assessment of the Neuropsychological Status), AES (Apathy Evaluation Scale), IMI (intrinsic Motivation Inventory), SUS (system Usability Scale), RAVLT (Rey Auditory Verbal Learning Test), PRM (Pattern Recognition Memory test—immediate and delayed recognition), PASAT (Paced Auditory Serial Addition Test), SSP (Spatial Span), RVP (Rapid Visual Information Processing), and EBIQ (European Brain Injury Questionnaire).

## Data Availability

Not applicable.

## References

[1] Dewan M.C., Rattani A., Gupta S., Baticulon R.E., Hung Y.C., Punchak M., Agrawal A., Adeleye A.O., Shrime M.G., Rubiano A.M. (2019). Estimating the global incidence of traumatic brain injury. J. Neurosurg..

[2] Mckee A.C., Daneshvar D.H. (2015). The neuropathology of traumatic brain injury. Handb. Clin. Neurol..

[3] Pavlovic D., Pekic S., Stojanovic M., Popovic V. (2019). Traumatic brain injury: Neuropathological, neurocognitive and neurobehavioral sequelae. Pituitary.

[4] De Luca R., Calabrò R.S., Bramanti P. (2018). Cognitive rehabilitation after severe acquired brain injury: Current evidence and future directions. Neuropsychol. Rehabil..

[5] Bland D.C., Zampieri C., Damiano D.L. (2011). Effectiveness of physical therapy for improving gait and balance in individuals with traumatic brain injury: A systematic review. Brain Inj..

[6] Gassert R., Dietz V. (2018). Rehabilitation robots for the treatment of sensorimotor deficits: A neurophysiological perspective. J. Neuroeng. Rehabil..

[7] Calabrò R.S., Cacciola A., Bertè F., Manuli A., Leo A., Bramanti A., Naro A., Milardi D., Bramanti P. (2016). Robotic gait rehabilitation and substitution devices in neurological disorders: Where are we now?. Neurol. Sci..

[8] Ferreira F.M.R.M., Chaves M.E.A., Oliveira V.C., Martins J.S.R., Vimieiro C.B.S., Van Petten A.M.V.N. (2021). Effect of Robot-Assisted Therapy on Participation of People with Limited Upper Limb Functioning: A Systematic Review with GRADE Recommendations. Occup. Ther. Int..

[9] Maggio M.G., De Luca R., Molonia F., Porcari B., Destro M., Casella C., Salvati R., Bramanti P., Calabrò R.S. (2019). Cognitive rehabilitation in patients with traumatic brain injury: A narrative review on the emerging use of virtual reality. J. Clin. Neurosci..

[10] Shin H., Kim K. (2015). Virtual reality for cognitive rehabilitation after brain injury: A systematic review. J. Phys. Ther. Sci..

[11] Oberholzer M., Müri R.M. (2019). Neurorehabilitation of Traumatic Brain Injury (TBI): A Clinical Review. Med. Sci..

[12] De Luca R., Bonanno M., Vermiglio G., Trombetta G., Andidero E., Caminiti A., Pollicino P., Rifici C., Calabrò R.S. (2022). Robotic Verticalization plus Music Therapy in Chronic Disorders of Consciousness: Promising Results from a Pilot Study. Brain Sci..

[13] Nudo R.J. (2011). Neural bases of recovery after brain injury. J. Commun. Disord..

[14] Braun R.G. (2021). Wittenberg GF. Motor Recovery: How Rehabilitation Techniques and Technologies Can Enhance Recovery and Neuroplasticity. Semin. Neurol..

[15] Semprini M., Laffranchi M., Sanguineti V., Avanzino L., De Icco R., De Michieli L., Chiappalone M. (2018). Technological Approaches for Neurorehabilitation: From Robotic Devices to Brain Stimulation and Beyond. Front. Neurol..

[16] Calabrò R.S., Müller-Eising C., Diliberti M.L., Manuli A., Parrinello F., Rao G., Barone V., Civello T. (2020). Who Will Pay for Robotic Rehabilitation? The Growing Need for a Cost-effectiveness Analysis. Inno. Clin. Neurosci..

[17] Lo A.C., Guarino P.D., Richards L.G., Haselkorn J.K., Wittenberg G.F., Federman D.G., Ringer R.J., Wagner T.H., Krebs H.I., Volpe B.T. (2011). An economic analysis of robot-assisted therapy for long-term upper-limb impairment after stroke. Stroke.

[18] Lo K., Stephenson M., Lockwood C. (2019). The economic cost of robotic rehabilitation for adult stroke patients: A systematic review. JBI Database System Rev. Implement Rep..

[19] Nascimento A.S., Fagundes C.V., Mendes F.A.D.S., Leal J.C. (2021). Effectiveness of Virtual Reality Rehabilitation in Persons with Multiple Sclerosis: A Systematic Review and Meta-analysis of Randomised Controlled Trials. Mult. Scler. Relat. Disord..

[20] Truijen S., Abdullahi A., Bijsterbosch D., van Zoest E., Conijn M., Wang Y., Struyf N., Saeys W. (2022). Effect of home-based virtual reality training and telerehabilitation on balance in individuals with Parkinson disease, multiple sclerosis, and stroke: A systematic review and meta-analysis. Neurol. Sci..

[21] Mehrholz J., Thomas S., Kugler J., Pohl M., Elsner B. (2020). Electromechanical-assisted training for walking after stroke. Cochrane Database Syst. Rev..

[22] Moher D., Shamseer L., Clarke M., Ghersi D., Liberati A., Petticrew M., Shekelle P., Stewart L.A. (2015). PRISMA-P Group. Preferred reporting items for systematic review and meta-analysis protocols (PRISMA-P) 2015 statement. Syst. Rev..

[23] De Morton N.A. (2009). The PEDro scale is a valid measure of the methodological quality of clinical trials: A demographic study. Aust. J. Physiother..

[24] Taveggia G., Ragusa I., Trani V., Cuva D., Angeretti C., Fontanella M., Panciani P.P., Borboni A. (2015). Robotic tilt table reduces the occurrence of orthostatic hypotension over time in vegetative states. Int. J. Rehabil. Res..

[25] Esquenazi A., Lee S., Wikoff A., Packel A., Toczylowski T., Feeley J. (2017). A Comparison of Locomotor Therapy Interventions: Partial-Body Weight-Supported Treadmill, Lokomat, and G-EO Training in People With Traumatic Brain Injury. PMR.

[26] Williams K., Christenbury J., Niemeier J.P., Newman M., Pinto S. (2020). Is Robotic Gait Training Feasible in Adults With Disorders of Consciousness?. J. Head Trauma Rehabil..

[27] Esquenazi A., Lee S., Packel A.T., Braitman L. (2013). A randomized comparative study of manually assisted versus robotic-assisted body weight supported treadmill training in persons with a traumatic brain injury. PMR.

[28] Cuthbert J.P., Staniszewski K., Hays K., Gerber D., Natale A., O’Dell D. (2014). Virtual reality-based therapy for the treatment of balance deficits in patients receiving inpatient rehabilitation for traumatic brain injury. Brain Inj..

[29] Ustinova K.I., Perkins J., Leonard W.A., Hausbeck C.J. (2014). Virtual reality game-based therapy for treatment of postural and co-ordination abnormalities secondary to TBI: A pilot study. Brain Inj..

[30] Tefertiller C., Hays K., Natale A., O’Dell D., Ketchum J., Sevigny M., Eagye C.B., Philippus A., Harrison-Felix C. (2019). Results From a Randomized Controlled Trial to Address Balance Deficits After Traumatic Brain Injury. Arch. Phys. Med. Rehabil..

[31] Corallo F., Maresca G., Formica C., Bonanno L., Bramanti A., Parasporo N., Giambò F.M., De Cola M.C., Lo Buono V. (2022). Humanoid Robot Use in Cognitive Rehabilitation of Patients with Severe Brain Injury: A Pilot Study. J. Clin. Med..

[32] Dvorkin A.Y., Ramaiya M., Larson E.B., Zollman F.S., Hsu N., Pacini S., Shah A., Patton J.L. (2013). A “virtually minimal” visuo-haptic training of attention in severe traumatic brain injury. J. Neuroeng. Rehabil..

[33] De Luca R., Bonanno M., Rifici C., Pollicino P., Caminiti A., Morone G., Calabrò R.S. (2022). Does Non-Immersive Virtual Reality Improve Attention Processes in Severe Traumatic Brain Injury? Encouraging Data from a Pilot Study. Brain Sci..

[34] Alashram A.R., Annino G., Padua E., Romagnoli C., Mercuri N.B. (2019). Cognitive rehabilitation post traumatic brain injury: A systematic review for emerging use of virtual reality technology. J. Clin. Neurosci..

[35] Aulisio M.C., Han D.Y., Glueck A.C. (2020). Virtual reality gaming as a neurorehabilitation tool for brain injuries in adults: A systematic review. Brain Inj..

[36] Ettenhofer M.L., Guise B., Brandler B., Bittner K., Gimbel S.I., Cordero E., Nelson Schmitt S., Williams K., Cox D., Roy M.J. (2019). Neurocognitive Driving Rehabilitation in Virtual Environments (NeuroDRIVE): A pilot clinical trial for chronic traumatic brain injury. NeuroRehabilitation.

[37] Jacoby M., Averbuch S., Sacher Y., Katz N., Weiss P.L., Kizony R. (2013). Effectiveness of executive functions training within a virtual supermarket for adults with traumatic brain injury: A pilot study. IEEE Trans. Neural. Syst. Rehabil. Eng..

[38] De Luca R., Maggio M.G., Maresca G., Latella D., Cannavò A., Sciarrone F., Lo Voi E., Accorinti M., Bramanti P., Calabrò R.S. (2019). Improving Cognitive Function after Traumatic Brain Injury: A Clinical Trial on the Potential Use of the Semi-Immersive Virtual Reality. Behav. Neurol..

[39] Lebowitz M.S., Dams-O’Connor K., Cantor J.B. (2012). Feasibility of computerized brain plasticity-based cognitive training after traumatic brain injury. J. Rehabil. Res. Dev..

[40] Zickefoose S., Hux K., Brown J., Wulf K. (2013). Let the games begin: A preliminary study using attention process training-3 and Lumosity brain games to remediate attention deficits following traumatic brain injury. Brain Inj..

[41] Ownsworth T., Arnautovska U., Beadle E., Shum D.H.K., Moyle W. (2018). Efficacy of Telerehabilitation for Adults With Traumatic Brain Injury: A Systematic Review. J. Head Trauma Rehabil..

[42] De Luca R., Maggio M.G., Naro A., Portaro S., Cannavò A., Calabrò R.S. (2020). Can patients with severe traumatic brain injury be trained with cognitive telerehabilitation? An inpatient feasibility and usability study. J. Clin. Neurosci..

[43] Raso M.G., Arcuri F., Liperoti S., Mercurio L., Mauro A., Cusato F., Romania L., Serra S., Pignolo L., Tonin P. (2021). Telemonitoring of Patients With Chronic Traumatic Brain Injury: A Pilot Study. Front. Neurol..

[44] Kang E.K., Kim D.Y., Paik N.J. (2012). Transcranial direct current stimulation of the left prefrontal cortex improves attention in patients with traumatic brain injury: A pilot study. J. Rehabil. Med..

[45] Hara T., Shanmugalingam A., McIntyre A., Burhan A.M. (2021). The Effect of Non-Invasive Brain Stimulation (NIBS) on Executive Functioning, Attention and Memory in Rehabilitation Patients with Traumatic Brain Injury: A Systematic Review. Diagnostics.

[46] Sacco K., Galetto V., Dimitri D., Geda E., Perotti F., Zettin M., Geminiani G.C. (2016). Concomitant use of transcranial direct current stimulation and computer-assisted training for the rehabilitation of attention in traumatic brain injured patients: Behavioral and neuroimaging results. Front. Behav. Neurosci.

[47] Ulam F., Shelton C., Richards L., Davis L., Hunter B., Fregni F., Higgins K. (2015). Cumulative effects of transcranial direct current stimulation on EEG oscillations and attention/working memory during subacute neurorehabilitation of traumatic brain injury. Clin. Neurophysiol..

[48] Leśniak M., Polanowska K., Seniów J., Członkowska A. (2014). Effects of repeated anodal tDCS coupled with cognitive training for patients with severe traumatic brain injury. J. Head Trauma Rehabil..

[49] Riener R., Lünenburger L., Colombo G. (2006). Human-centered robotics applied to gait training and assessment. J. Rehabil. Res. Dev..

[50] Lünenburger L., Colombo G., Riener R., Dietz V. (2007). Biofeedback in gait training with the robotic orthosis Lokomat. Conf. Proc. IEEE Eng. Med. Biol. Soc..

[51] Santamaria V., Luna T., Khan M., Agrawal S. (2020). The robotic Trunk-Support-Trainer (TruST) to measure and increase postural workspace during sitting in people with spinal cord injury. Spinal Cord Ser. Cases.

[52] Hornby T.G., Reisman D.S., Ward I.G., Scheets P.L., Miller A., Haddad D., Fox E.J., Fritz N.E., Hawkins K., Henderson C.E. (2020). Clinical Practice Guideline to Improve Locomotor Function Following Chronic Stroke, Incomplete Spinal Cord Injury, and Brain Injury. J. Neurol. Phys. Ther..

[53] Turolla A., Venneri A., Farina D., Cagnin A., Cheung V. (2018). Rehabilitation Induced Neural Plasticity after Acquired Brain Injury. Neural. Plast..

[54] Retel Helmrich I., Lingsma H.F., Turgeon A.F., Yamal J.M., Steyerberg E.W. (2021). Prognostic Research in Traumatic Brain Injury: Markers, Modeling, and Methodological Principles. J. Neurotrauma.

[55] Assad-Uz-Zaman M., Rasedul Islam M., Miah S., Rahman M.H. (2019). NAO robot for cooperative rehabilitation training. J. Rehabil. Assist. Technol. Eng..

[56] Subramanian S.K., Fountain M.K., Hood A.F., Verduzco-Gutierrez M. (2022). Upper Limb Motor Improvement after Traumatic Brain Injury: Systematic Review of Interventions. Neurorehabil. Neural. Repair.

[57] Calabrò R.S., Naro A., Russo M., Leo A., De Luca R., Balletta T., Buda A., La Rosa G., Bramanti A., Bramanti P. (2017). The role of virtual reality in improving motor performance as revealed by EEG: A randomized clinical trial. J. Neuroeng. Rehabil..

[58] Hao J., Xie H., Harp K., Chen Z., Siu K.C. (2022). Effects of Virtual Reality Intervention on Neural Plasticity in Stroke Rehabilitation: A Systematic Review. Arch. Phys. Med. Rehabil..

[59] Highland K.B., Kruger S.E., Roy M.J. (2015). If You Build It, They Will Come, But What Will Wounded Warriors Experience? Presence in the CAREN. Stud. Health Technol. Inform..

[60] Sessoms P.H., Gottshall K.R., Collins J.D., Markham A.E., Service K.A., Reini S.A. (2015). Improvements in gait speed and weight shift of persons with traumatic brain injury and vestibular dysfunction using a virtual reality computer-assisted rehabilitation environment. Mil. Med..

[61] Lippa S.M., Rosen K.B., Delpy K.B., Pape M.M., Kruger S.E. (2021). Overground and Virtual Reality Gait Speed Are Associated With Atypical Symptom Reporting in Active Duty Service Members With a History of Mild to Moderate Traumatic Brain Injury. J. Head Trauma Rehabil..

[62] Fetta J., Starkweather A., Gill J.M. (2017). Computer-Based Cognitive Rehabilitation Interventions for Traumatic Brain Injury: A Critical Review of the Literature. J. Neurosci. Nurs..

[63] Subbarao B.S., Stokke J., Martin S.J. (2021). Telerehabilitation in Acquired Brain Injury. Phys. Med. Rehabil. Clin. N. Am..

[64] Calabrò R.S., Bramanti A., Garzon M., Celesti A., Russo M., Portaro S., Naro A., Manuli A., Tonin P., Bramanti P. (2018). Telerehabilitation in individuals with severe acquired brain injury: Rationale, study design, and methodology. Medicine.

[65] Bramanti A., Manuli A., Calabrò R.S. (2018). Stroke Telerehabilitation in Sicily: A Cost-Effective Approach to Reduce Disability?. Innov. Clin. Neurosci..

[66] Lindberg B., Nilsson C., Zotterman D., Söderberg S., Skär L. (2013). Using Information and Communication Technology in Home Care for Communication between Patients, Family Members, and Healthcare Professionals: A Systematic Review. Int. J. Telemed. Appl..

[67] Stagg C.J., Lin R.L., Mezue M., Segerdahl A., Kong Y., Xie J., Tracey I. (2013). Widespread modulation of cerebral perfusion induced during and after transcranial direct current stimulation applied to the left dorsolateral prefrontal cortex. J. Neurosci..

[68] De Munter L., Polinder S., Havermans R.J.M., Steyerberg E.W., de Jongh M.A.C. (2021). Prognostic factors for recovery of health status after injury: A prospective multicentre cohort study. BMJ Open.

